# Supplementary material for: Sustainable open data ecosystems through data quality, governance, and infrastructure: Unlocking social, political and economic value
Source: Open Res Eur. 2025 Sep 23;5:295. [Version 1] doi: 10.12688/openreseurope.21212.1 (PMC12531620; doi:10.12688/openreseurope.21212.1)
Supplement: Peer review history

# Reviewer report: Reviewer response for version 1

**DOI:** 10.21956/openreseurope.22945.r61290

**Published:** 2025-11-12

**Authors:** Sophie Weerts, Clarissa Valli Buttow

**Affiliations:** 1 University of Lausanne, Lausanne, Switzerland; 2 Swiss Graduate School of Public Administration, Universite de Lausanne Faculte de Droit des sciences criminelles et d'administration publique, Lausanne, Vaud, Switzerland

This publication is an open letter that summarises the key contributions of the ODECO project consortium to sustainable open data ecosystems. It provides nine practical recommendations, which are categorised into three clusters: data quality, wide data re-use, and open data infrastructure. In this regard, the paper aims to highlight topics that public authorities could develop to contribute to sustainable open data ecosystems. The open letter is logically organised into five sections: an introduction; a theoretical framework; a summary of the findings; a presentation of the recommendations; and a conclusion. To formulate their nine recommendations, the authors conducted a narrative review of the ODECO reports.

 The open letter offers an interesting roadmap based on extensive reports. However, reviewing this document raises several points that may help the authors clarify their ideas and emphasise the practical outcomes of this important research project.  

 In the introduction, the authors could clarify what a sustainable data ecosystem means, why current ecosystems are not sustainable, and the role that open data could play in making them sustainable.  We understand that the ODECO partners adopted a common starting point that more data is beneficial for society (its government and the economy) — useful for uniting researchers with different backgrounds. However, we believe that researchers should clarify this epistemological standpoint and the fact that it aligns with the narrative and agenda of the European Union. Furthermore, the open letter should indicate which period covers the research project, what bring their recommendations in comparison to those formulated in European reports (High-value Datasets Best practice (2024) and Open data maturity report (2023)).

 On a more detailed level, the authors indicate that, on the production side of OGD, public administrations often make opaque decisions about which information to release as open data, and they provide references dating from 2014 and 2016. We wonder if the authors should not be more nuanced. On one side, European data regulation has substantially evolved since 2016. On the other side, data are always processed and there will probably never be a perfectly uniform process, as any action in the data processing will require human intervention. The authors also point out that one issue is the lack of public access to internet connectivity. Their argument would be strengthened if they provided data to support this statement. 

 In the section on "open data policy and regulation in the EU", the authors do not provide a summary of the current policy and regulatory context. Perhaps reports included findings that highlight the current regulatory framework in the EU, which has evolved substantially with the Open Data Directive and the adoption of other provisions such as in the Digital Services Act, which require access to social media data. Furthermore, the authors state that the EU has identified six categories of high-value datasets, but only list five.

 In the same section, the authors emphasise the closure logic regarding data and the need to counterbalance the economic trend. The authors could provide an example of open and free government data being used by commercial actors to develop a product or service. Elsewhere in the letter, they emphasise the need to adopt a critical approach to data and its infrastructure. However, a few points in the critical literature seem to be missing (such as the lack of evidence on the results of OGD efforts, or the absence of a discussion of technological consequences and democratic oversight of data reuse). 

 The recommendations section could be developed to provide policymakers with the same level of granular information. Regarding data quality, for example, the first two recommendations on discoverability and metadata are rather abstract and vague compared to the recommendation on ongoing engagement and participation. The cluster-wide data reuse, gathering beyond OGD, data literacy, and 'mind the gaps' recommendations sound a bit artificial. The authors could clarify how the 'Mind the Gaps' recommendation fits within the 'Cluster-wide Data Reuse' initiative. Finally, the last cluster contains a misunderstanding regarding the roles of public administrations and governments. Many aspects mentioned under 'public administration investments' depend on public authorities' decisions (e.g. building new infrastructure or funding new projects). For this reason, the authors should adapt their terminology.

Where applicable, are recommendations and next steps explained clearly for others to follow? (Please consider whether others in the research community would be able to implement guidelines or recommendations and/or constructively engage in the debate)

Partly

Does the article adequately reference differing views and opinions?

Partly

Are all factual statements correct, and are statements and arguments made adequately supported by citations?

Partly

Is the rationale for the Open Letter provided in sufficient detail? (Please consider whether existing challenges in the field are outlined clearly and whether the purpose of the letter is explained)

Partly

Is the Open Letter written in accessible language? (Please consider whether all subject-specific terms, concepts and abbreviations are explained)

Yes

Reviewer Expertise:

Law and regulation studies

We confirm that we have read this submission and believe that we have an appropriate level of expertise to confirm that it is of an acceptable scientific standard, however we have significant reservations, as outlined above.

# Reviewer report: Reviewer response for version 1

**DOI:** 10.21956/openreseurope.22945.r61294

**Published:** 2025-10-29

**Authors:** Enric Senabre Hidalgo

**Affiliations:** 1 Universitat de Barcelona, Barcelona, Spain

The manuscript of this open letter I think is well-structured, insightful and a great example of derivative work from this EU project, providing 9 key recommendations in a well-described and research-based approach. From my experience and opinion on the topic of open data I consider it's ready for publication with minor considerations. While important aspects like the focus on sustainability, equity and the CARE principles is a significant strength, among others, here are however a series of minor suggestions in order of importance:

 (1) My main formal consideration is that the nine evocative/metaphoric figures accompanying each recommendation don't seem to add value and may detract from the letter's attractiveness due to their lack of visual coherence. A minor recommendation is to either remove the visuals entirely or maybe commission a set of same-style, coherent visuals.

 (2) Although the letter mentions AI in relation to open data in different subsections, the discussion could be more critical and reflective of the current fast-paced development of LLMs, agents and AI platforms that extract value from open data, potentially disrupting community-led efforts (like it's happening in the moment of writing this with Wikipedia or citizen science platforms). This could be strengthened or more developed in the discussion section. Also, the brief comment on "responsible use of AI" in Section 7 (Usability) and the lack of reflection on data extractivism in Section 8 (Interoperability) could be expanded slightly with a sentence or two for greater clarity.

 (3) I also consider a little more detail is needed on the narrative review before the 9 thematic categories. The current description is brief and lacks a little bit of more methodological details (e.g., who was involved, under which specific methodological process or step-by-step, like the thematic analysis process I guess was used). Expanding this section would enhance academic transparency.

 (4) Subtle improvements would be to develop more descriptive titles for the 9 recommendations, as some are currently too short and undescriptive, such as "2. Metadata" or "6. Mind the gap(s)", etc.

 (5) Regarding Section 6 precisely ("Mind the gap(s)"), the concept of "open data justice" could be explicitly connected to the previous concept of design justice and data feminism. Please consider including the following reference: Nasrin, S. (2023). New ways of activism: Design justice and data feminism. Social Movement Studies, 22(1), 140–144.

 (6) Finally, I also miss a possible reference in Section 9 (Public administrations investments) regarding the investment or promotion of open data cooperatives as a tangible public-commons partnership. Please consider also this reference: Bühler, M. M., Calzada, I., Cane, I., Jelinek, T., Kapoor, A., Mannan, M., ... & Zhu, J. (2023). Unlocking the power of digital commons: Data cooperatives as a pathway for data sovereign, innovative and equitable digital communities. Digital, 3(3), 146-171.

Where applicable, are recommendations and next steps explained clearly for others to follow? (Please consider whether others in the research community would be able to implement guidelines or recommendations and/or constructively engage in the debate)

Yes

Does the article adequately reference differing views and opinions?

Partly

Are all factual statements correct, and are statements and arguments made adequately supported by citations?

Yes

Is the rationale for the Open Letter provided in sufficient detail? (Please consider whether existing challenges in the field are outlined clearly and whether the purpose of the letter is explained)

Yes

Is the Open Letter written in accessible language? (Please consider whether all subject-specific terms, concepts and abbreviations are explained)

Yes

Reviewer Expertise:

Open science; Digital Commons; Social and Solidarity Economy

I confirm that I have read this submission and believe that I have an appropriate level of expertise to confirm that it is of an acceptable scientific standard.

## References

[1] : New ways of activism: design justice and data feminism. *Social Movement Studies* .2023;22(1) : 10.1080/14742837.2021.1967132 140-144 10.1080/14742837.2021.1967132

[2] : Unlocking the Power of Digital Commons: Data Cooperatives as a Pathway for Data Sovereign, Innovative and Equitable Digital Communities. *Digital* .2023;3(3) : 10.3390/digital3030011 146-171 10.3390/digital3030011

# Reviewer report: Reviewer response for version 1

**DOI:** 10.21956/openreseurope.22945.r61293

**Published:** 2025-10-16

**Authors:** Arwid Lund

**Affiliations:** 1 Södertörn University, Stockholm, Sweden

This open letter is a report that summarizes key findings of the research project ODECO (Open Data ECOsystems), a consortium within Marie Curie Action Innovative Training Networks. The project ran between 2021 – 2025 and developed a broad perspective on OD valuation including economic value generation *and* social valuation within a theoretical ecosystemic framework, focused on sustainability. The report is clearly structured with a state-of-art section on current research, a section  on the project and its methodology, and a final part with recommendations for building sustainable OD ecosystems that empowers all stakeholders. 

 The rationale for the report is clearly stated and the groundwork for the suggested recommendations are well-documented, factually correct and written in an accessible language. The report's recommendations are described in detail and clustered in three themes: data production and access, data reuse and the long-term perspective with a focus on infrastructural issues. 

 The report highlights and develops a very much needed critical perspective on the character of the *openness* of OD. Who provides the OD, for whom and for what purpose? Raising for example the issue of how to incentivize non-government actors to release open datasets.

 On the critical side, I would like to problematise the theoretical ecosystemic perspective. It works well to give a picture of the interconnectedness of all stakeholders. Stakeholders that are listed as “local government, regional/central government, non-specialist data users, journalists, students, non-profit organisations, companies, artificial users, and open data intermediaries”. The weakness I perceive is connected to how the ecosystem metaphor affects our understanding of the interconnectedness. The idea of an ecosystem assumes some kind of equilibrium and sustainability already from the start, and like the network metaphor it somewhat obscures systematic imbalances and power relations, for example from a critical political economic perspective (note for example the position of companies in the list above). The critique that results from the report is limited to proposing complementary forms of social valuation in relation to the structural and systematic economic exploitation and enclosure of OD that is driven by private non-governmental actors.

 On the positive side, the alternative visions of OD ecosystems are richly elaborated, especially by highlighting the importance of government support to commons-based projects. The report's recommendations invite all stakeholders to take constructive part in discussions.

Where applicable, are recommendations and next steps explained clearly for others to follow? (Please consider whether others in the research community would be able to implement guidelines or recommendations and/or constructively engage in the debate)

Yes

Does the article adequately reference differing views and opinions?

Yes

Are all factual statements correct, and are statements and arguments made adequately supported by citations?

Yes

Is the rationale for the Open Letter provided in sufficient detail? (Please consider whether existing challenges in the field are outlined clearly and whether the purpose of the letter is explained)

Yes

Is the Open Letter written in accessible language? (Please consider whether all subject-specific terms, concepts and abbreviations are explained)

Yes

Reviewer Expertise:

Open Science, Open Data, Open Governmental Data, Open Access, Open Source, Commons-Based Peer-Production, Digital Platforms

I confirm that I have read this submission and believe that I have an appropriate level of expertise to confirm that it is of an acceptable scientific standard.

# Reviewer report: Reviewer response for version 1

**DOI:** 10.21956/openreseurope.22945.r61289

**Published:** 2025-10-14

**Authors:** Mohsan Ali

**Affiliations:** 1 University of the Aegean, Samos, Greece

The report discusses the open data ecosystem perspectives and, based on the research group activities over the years, extracted recommendations for enabling and improving OD ecosystems in the long term. This letter is equally important for the research purpose and for the policy makers and other user groups in the open data multidisciplinary domain.

 In its current form, it is well organized and presents detailed information regarding open data ecosystem specificities.  

 Thanks

Where applicable, are recommendations and next steps explained clearly for others to follow? (Please consider whether others in the research community would be able to implement guidelines or recommendations and/or constructively engage in the debate)

Yes

Does the article adequately reference differing views and opinions?

Yes

Are all factual statements correct, and are statements and arguments made adequately supported by citations?

Yes

Is the rationale for the Open Letter provided in sufficient detail? (Please consider whether existing challenges in the field are outlined clearly and whether the purpose of the letter is explained)

Yes

Is the Open Letter written in accessible language? (Please consider whether all subject-specific terms, concepts and abbreviations are explained)

Yes

Reviewer Expertise:

Open Data Technical Interoperability

I confirm that I have read this submission and believe that I have an appropriate level of expertise to confirm that it is of an acceptable scientific standard.
